# Combinations of scalp acupuncture location for the treatment of post-stroke hemiparesis: A systematic review and *Apriori* algorithm-based association rule analysis

**DOI:** 10.3389/fnins.2022.956854

**Published:** 2022-08-05

**Authors:** Yu-Fang Wang, Wei-Yi Chen, Chang-Ti Lee, Yi-Ying Shen, Chou-Chin Lan, Guan-Ting Liu, Chan-Yen Kuo, Mao-Liang Chen, Po-Chun Hsieh

**Affiliations:** ^1^Department of Chinese Medicine, Taipei Tzu Chi Hospital, Buddhist Tzu Chi Medical Foundation, New Taipei City, Taiwan; ^2^Division of Pulmonary Medicine, Taipei Tzu Chi Hospital, Buddhist Tzu Chi Medical Foundation, New Taipei City, Taiwan; ^3^School of Medicine, Tzu-Chi University, Hualien, Taiwan; ^4^Department of Research, Taipei Tzu Chi Hospital, Buddhist Tzu Chi Medical Foundation, New Taipei City, Taiwan

**Keywords:** stroke, hemiparesis, scalp acupuncture, *Apriori* algorithm, association rule analysis

## Abstract

**Background:**

Post-stroke hemiparesis strongly affects stroke patients’ activities of daily living and health-related quality of life. Scalp acupuncture (SA) is reportedly beneficial for post-stroke hemiparesis. However, there is still no standard of SA for the treatment of post-stroke hemiparesis. *Apriori* algorithm-based association rule analysis is a kind of “if-then” rule-based machine learning method suitable for investigating the underlying rules of acupuncture point/location selections. This study aimed to investigate the core SA combinations for the treatment of post-stroke hemiparesis by using a systematic review and *Apriori* algorithm-based association rule analysis.

**Methods:**

We conducted a systematic review to include relevant randomized controlled trial (RCT) studies investigating the effects of SA treatment in treating patients with post-stroke hemiparesis, assessed by the Fugl-Meyer Assessment (FMA) score. We excluded studies using herbal medicine or manual acupuncture.

**Results:**

We extracted 33 SA locations from the 35 included RCT studies. The following SA styles were noted: International Standard Scalp Acupuncture (ISSA), WHO Standard Acupuncture Point Locations (SAPL), Zhu’s style SA, Jiao’s style SA, and Lin’s style SA. Sixty-one association rules were investigated based on the integrated SA location data.

**Conclusions:**

SAPL_GV20 (Baihui), SAPL_GV24 (Shenting), ISSA_MS6_i (ISSA Anterior Oblique Line of Vertex-Temporal, lesion-ipsilateral), ISSA_MS7_i (ISSA Posterior Oblique Line of Vertex-Temporal, lesion-ipsilateral), ISSA_PR (ISSA Parietal region, comprised of ISSA_MS5, ISSA_MS6, ISSA_MS7, ISSA_MS8, and ISSA_MS9), and SAPL_Ex.HN3 (Yintang) can be considered the core SA location combination for the treatment of post-stroke hemiparesis. We recommend a core SA combination for further animal studies, clinical trials, and treatment strategies.

## Introduction

Stroke is the second most common cause of mortality, leading also to disability in middle-aged and elderly populations worldwide, with the affected populations trending younger (Lindsay et al., 2019; GBD 2019 Diseases and Injuries Collaborators, 2020). Most stroke survivors suffer from sequelae, such as cognitive change, expression disorder, sensory disturbance, weakness, hemiparesis, and hemiplegia. The sequelae result in a decline of self-care ability and affect the patients’ activities of daily living (ADL) and health-related quality of life (HRQL) (Campbell et al., 2019). Hemiparesis is defined as complete or incomplete muscular weakness or paralysis affecting either side of the body, primarily affecting ADL and HRQL (Bindawas et al., 2017). Patients with hemiparesis show various symptoms depending on their stroke severity, such as muscle weakness or stiffness, muscle spasticity or permanently contracted muscle, difficulty walking or grabbing objects, poor balance, decreased movement precision, and lack of motor coordination (Bindawas et al., 2017). For post-stroke hemiparesis, physical therapy-based rehabilitation training management can improve upper limb movement ability, increase muscle strength, reduce limb pain, and improve quality of life (Huang et al., 2022).

Acupuncture therapy is considered beneficial for post-stroke rehabilitation, such as aphasia, insomnia, neuroplasticity, depression, and cognitive impairment (Yang et al., 2016). Scalp acupuncture (SA) is a modern acupuncture technique integrating traditional Chinese medicine acupuncture methods and the functionality of brain areas to stimulate different scalp locations (point, area, or zone) with needles (Liu et al., 2012). Scalp acupuncture is widely applied to treat central nervous system disorders, such as Parkinson’s disease (Lee et al., 2013), spinal cord injury (Widrin, 2018), and stroke (Liu et al., 2012; Wang et al., 2012; Huang et al., 2021; Zhang et al., 2021). Huang et al. (2021) reported that SA combined with conventional treatment (rehabilitation training and conventional medication) shows significant differences in motor function (by Fugl-Myer Assessment [FMA] scores) compared to conventional treatment in patients with post-stroke hemiparesis (regardless of brain infarction or intracerebral hemorrhage) after 1- and 3-month treatment courses. In clinical practice, there are various types of SA, such as the WHO Standard Acupuncture Point Locations (SAPL), (World Health Organization Regional Office for the Western Pacific, 2009) International Standard Scalp Acupuncture (ISSA), (World Health Organization Regional Office for the Western Pacific, 1991) Zhu’s style SA (Zhu, 2007) Jiao’s style SA (Jiao, 1997), and Lin’s style SA (Wang, 2019). However, there is still no standard of SA for the treatment of post-stroke hemiparesis.

*Apriori* algorithm-based association rule analysis is a kind of “if-then” rule-based machine learning method for discovering interesting relations between variables in databases to identify frequent individual item sets (Bayardo and Agrawal, 1999). Since acupuncture therapy is clinically practiced based on acupuncture point combinations, association rules analysis is suitable to investigate the underlying rules of acupuncture point selections. The previous studies using data mining methods revealed the core combinations of acupuncture points in treating various neurological disorders, such as Parkinson’s disease (Li et al., 2020a), vascular dementia (Feng et al., 2015), and Alzheimer’s disease (Yu et al., 2018).

This study aimed to investigate the core SA combinations for the treatment of post-stroke hemiparesis by using a systematic review and *Apriori* algorithm-based association rule analysis.

## Materials and methods

### Search strategy

We conducted a systematic review that followed the Preferred Reporting Items for Systematic Reviews and Meta-analysis (PRISMA) guidelines (Page et al., 2021). Five major electronic databases were searched, such as three English databases (PubMed, Embase, and the Cochrane library) and two Chinese databases (Airiti Library and China National Knowledge Infrastructure) from initiation until April 2022 without language limitation. The keywords used for the search were: stroke and scalp acupuncture in English and Chinese. The detailed search strategies were presented in Supplementary Table 1. Two reviewers (YFW and WYC) independently reviewed the titles and abstracts of the identified articles. Discrepancies or issues between the reviewers were resolved by consulting with a third reviewer (PCH) as an arbiter.

### Study selection criteria

The target population was patients with post-stroke hemiparesis. The included studies were required to have a treatment arm using SA and use FMA, a stroke-specific, performance-based index, as motor function assessment. The FMA index is a clinical and research tool for evaluating changes in motor impairment following hemiplegic stroke patients (Gladstone et al., 2002). The study design was restricted to randomized controlled trials (RCTs). We excluded studies using herbal medicine or manual acupuncture.

### Data extraction

A predetermined form was used by two reviewers (YFW and WYC) independently for data extraction with the following information: author, year, the sample size in the SA and control groups, mean age of the patients, sex, SA treatment details (such as retention time, treatment frequency, and total treatment course), and SA locations. We extracted the SA locations that were used in the included studies to create binary data for analysis (Supplementary Table 2). Definitions of the SA locations were as per the SAPL, (World Health Organization Regional Office for the Western Pacific, 2009) ISSA, (World Health Organization Regional Office for the Western Pacific, 1991) Zhu’s style SA, (Zhu, 2007) Jiao’s style SA, (Jiao, 1997) Lin’s style SA, (Wang, 2019), and other kinds of SA style.

### Data analysis

This study processed the *Apriori* algorithm-based association rule analysis (Bayardo and Agrawal, 1999) and plotting using software R (version 3.4.3 for Windows). The procedure can be conveniently fitted using the R package “rules”, while the visualizing association rules can be directly fitted using the R package “arulesViz”. Basically, an *Apriori* algorithm-based association rule consists of an antecedent and a consequent, both of which are a list of items. It is important to note that the implication here is co-occurrence and not causality. Item set is the list of all the items in the antecedent and the consequent for a given rule. The association rule analysis involves three main kernel values: contained support, confidence, and lift. Mathematically, support is the fraction of the total number of transactions in which the item set occurs. Technically, confidence is the conditional probability of occurrence of the consequent, given the antecedent. The lift value of an association rule is the ratio of joint probability (of an antecedent and a consequent) and the product of their marginal probabilities. In this study, we conducted the analysis of the top 10 frequently used association rules, and the minimum requirements were determined as support degree ≥10% and confidence ≥80%. Furthermore, we reported the association rules according to descending support and confidence and lift values corresponding to the support of the association rules.

### Quality assessment of the included studies

Two reviewers (YFW and WYC) independently assessed the methodological quality of the included studies using the Quality Assessment of Controlled Intervention Studies tool (developed by the National Heart, Lung, and Blood Institute, National Institutes of Health [NIH], 2021) and discrepancies were resolved by the third reviewer (PCH).

## Results

### Study identification

**FIGURE 1:**
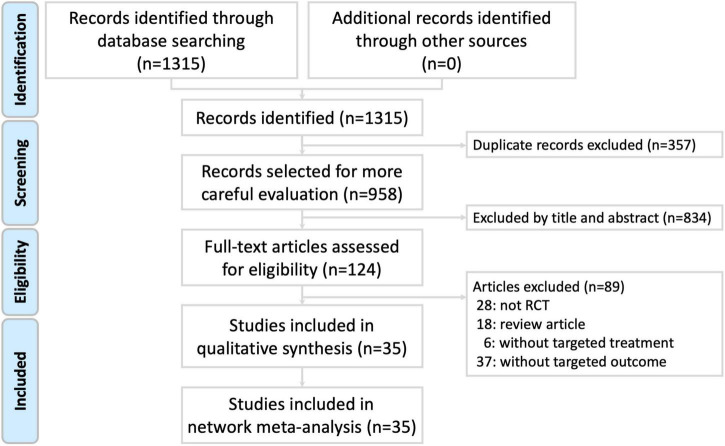
Study flow diagram.

A total of 1,315 studies were identified by the search terms. We eliminated the duplicate studies, excluded studies by reading the titles and abstracts, and conducted full-text evaluations for eligibility. Finally, 35 eligible RCT studies (Xie et al., 2007; Li and Zheng, 2009; Ma et al., 2010, 2019; Fu et al., 2011; Qin et al., 2013; Kong et al., 2014; Qin, 2015; Tan and Yu, 2015; Xu et al., 2015, 2018; Zhang et al., 2015, 2021; Chen et al., 2016; Dou et al., 2016; Liu et al., 2016; Pan et al., 2017; Wang et al., 2017, 2020; Yang et al., 2017; Yin, 2017; Hu, 2018; Qi et al., 2018; Xiao, 2018; Chen and Jiang, 2019; Hu and Yang, 2019; Li and Zhang, 2019; Sun, 2019; Ye et al., 2019; Zhang H.-L. et al., 2019; Zhang X.-Y. et al., 2019; Zhao and Yang, 2019; Zhu et al., 2019; Xiong et al., 2020; Yin et al., 2020) were included for the *Apriori* algorithm-based association rule analysis. The PRISMA study flow diagram is presented in Figure 1. The List of excluded articles with reasons after the full-text evaluation is presented in Supplementary Table 3.

### Study characteristics

**FIGURE 2:**
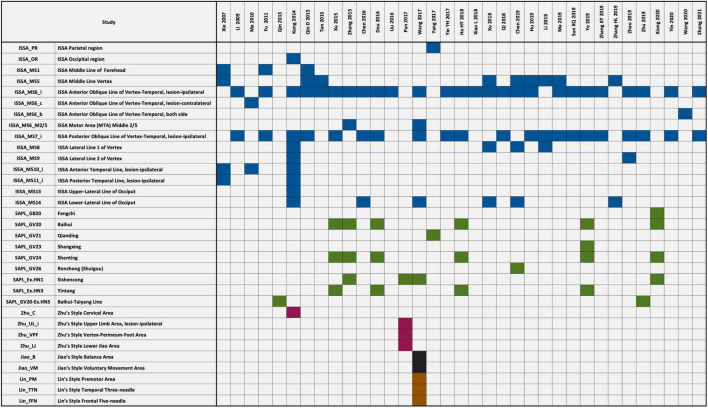
Scalp acupuncture (SA) locations used in the included studies. The full names and the abbreviations of the scalp acupuncture (SA) location used in the association rule analysis are listed in the left panel. The SA locations used in the included studies are shown with filled color in the right panel. Blue: International Standard Scalp Acupuncture (ISSA). Green: WHO Standard Acupuncture Point Locations (SAPL). Pink: Zhu’s style SA. Black: Jiao’s style SA. Brown: Lin’s style SA.

The summary of the characteristics in the included studies is presented in Table 1. The summary of the SA locations (such as SA location full name and code for analysis) used in the included studies is presented in Figure 2.

**TABLE 1:** Summary of the included studies.

Author, year	Sample size (SA/C)	Mean age, mean ± SD		Male, *n* (%)		SA treatment detail		
		SA group	Control Group	SA group	Control group	Retention (min)	Frequency (time/week)	Course (week)
Xie et al., 2007	41/39	53.0 ± 9.3	56.5 ± 6.4	24 (58.5%)	22 (56.4%)	30	3–4	12
Li and Zheng, 2009	45/45	61 ± 6	63 ± 6	24 (53.3%)	27 (60.0%)	45–60	5	6
Ma et al., 2010	15/15	62.1 ± 0.5	61.4 ± 0.2	8 (53.3%)	9 (60.0%)	30	7	3
Fu et al., 2011	32/32	38–81	38–81	37 (57.8%)	37 (57.8%)	360	6	8
Qin et al., 2013	20/20	60.0 ± 6.4	61.0 ± 7.1	12 (60.0%)	11 (55.0%)	30	3	12
Kong et al., 2014	33/33	60.0 ± 9.7	62.0 ± 8.9	23 (69.7%)	21 (63.6%)	180–300	7	12
Qin, 2015	58/58	64.7 ± 5.7	65.3 ± 7.9	39 (67.2%)	36 (62.1%)	30	3–4	12
Tan and Yu, 2015	80/80	58.4 ± 10.5	59.3 ± 11.5	40 (50.0%)	42 (52.5%)	120	7	4
Xu et al., 2015	60/60	58.3 ± 11.2	61.7 ± 9.1	45 (75.0%)	42 (70.0%)	NR	6	4
Zhang et al., 2015	30/30	63.0 ± 4.0	62.0 ± 5.0	16 (53.3%)	15 (50.0%)	120–180	NR	4
Chen et al., 2016	29/30	56.1 ± 6.5	55.3 ± 5.9	18 (62.1%)	16 (53.3%)	60	5	4
Dou et al., 2016	33/33	58.7 ± 2.4	59.8 ± 2.8	23 (69.7%)	22 (66.7%)	NR	6	4
Liu et al., 2016	30/30	58.6 ± 4.9	60.5 ± 3.9	17 (56.7%)	20 (66.7%)	60	5	4
Pan et al., 2017	53/53	64.1 ± 9.7	62.9 ± 10.8	29 (54.7%)	28 (52.8%)	480	7	4
Wang et al., 2017	60/60	62.0 ± 10.0	63.0 ± 8.0	49 (81.7%)	50 (83.3%)	60	5	4
Yang et al., 2017	18/18	47.0 ± 8.4	49.0 ± 8.2	10 (55.6%)	10 (55.6%)	360	5	4
Yin, 2017	50/50	65.2 ± 3.4	64.5 ± 3.2	28 (56.0%)	26 (52.0%)	30	6	8
Hu, 2018	56/56	64.7 ± 6.1	64.7 ± 6.1	61 (54.5%)	61 (54.5%)	60	7	8
Xiao, 2018	54/54	57.1 ± 9.7	57.1 ± 9.7	63 (58.3%)	63 (58.3%)	60	5	4
Xu et al., 2018	25/25	58.4 ± 8.3	58.3 ± 8.0	12 (48.0%)	13 (52.0%)	30	5	4
Chen and Jiang, 2019	20/20	62.5 ± 11.7	68.6 ± 4.8	12 (60.0%)	11 (55.0%)	40	5	6
Hu and Yang, 2019	34/34	62.0 ± 10.0	62.0 ± 8.0	19 (55.9%)	17 (50.0%)	360	6	4
Li and Zhang, 2019	38/40	68.0 ± 8.0	68.7 ± 8.3	22 (57.9%)	23 (57.5%)	20–30	5	6
Ma et al., 2019	20/20	67.2 ± 5.7	65.4 ± 4.8	10 (50.0%)	9 (45.0%)	25*2	6	4
Sun, 2019	45/45	63.3 ± 2.7	63.2 ± 2.2	23 (51.1%)	22 (48.9%)	30	5	24
Ye et al., 2019	46/46	65.1 ± 7.3	65.0 ± 7.1	33 (71.7%)	34 (73.9%)	60	5	8
Zhang X.-Y. et al., 2019	40/40	54.5 ± 4.4	52.4 ± 12.1	23 (57.5%)	21 (52.5%)	30	6	6
Zhang H.-L. et al., 2019	34/34	64.6 ± 9.3	64.0 ± 9.6	18 (52.9%)	17 (50.0%)	NR	NR	12
Zhao and Yang, 2019	45/45	60.8 ± 7.4	59.9 ± 8.6	27 (60.0%)	29 (64.4%)	30	7	2
Zhu et al., 2019	40/40	49.0 ± 3.7	54.0 ± 1.9	27 (67.5%)	17 (42.5%)	30	3–4	8
Qi et al., 2018	30/30	64.0 ± 11.0	65.0 ± 9.0	19 (63.3%)	14 (46.7%)	30	5	24
Xiong et al., 2020	35/35	63.0 ± 7.2	65.3 ± 8.5	20 (57.1%)	17 (48.6%)	180–240	6	8
Yin et al., 2020	26/25	55.0 ± 10.0	52.0 ± 11.0	17 (65.4%)	17 (68.0%)	40	5	8
Wang et al., 2020	58/58	62.4 ± 9.0	59.5 ± 8.9	39 (67.2%)	41 (70.7%)	30	6	2
Zhang et al., 2021	70/72	57.4 ± 15.5	55.5 ± 17.2	35 (50.0%)	40 (55.6%)	30	6	8

C, control group; NR, not reported; SA, scalp acupuncture; SD, standard deviation.

### Distribution of the acupoint

**FIGURE 3:**
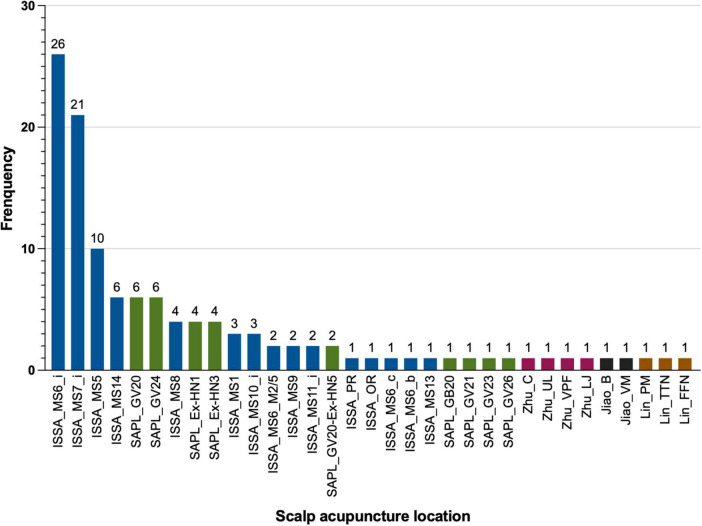
Distribution of the scalp acupuncture (SA) locations used in the included studies. The full names of the abbreviations of the SA locations are listed in Figure 1. Blue: International Standard Scalp Acupuncture (ISSA). Green: WHO Standard Acupuncture Point Locations (SAPL). Pink: Zhu’s style SA. Black: Jiao’s style SA. Brown: Lin’s style SA.

We extracted 33 SA locations from the 35 included RCT studies. The distribution details of the acupoint frequency are shown in Figure 3. The top 11 frequently selected acupoints were ISSA_MS6_i, ISSA_MS7_i, ISSA_MS5, ISSA_MS14, SAPL_GV20, SAPL_GV24, ISSA_MS8, SAPL_EX-HN1, SAPL_EX-HN3, ISSA_MS1, and ISSA_MS10_i. These SA locations were frequently used in treating stroke-related symptoms and rehabilitation in clinical practice.

### *Apriori* algorithm-based association rule analysis for the itemsets of Scalp acupuncture location combinations

**FIGURE 4:**
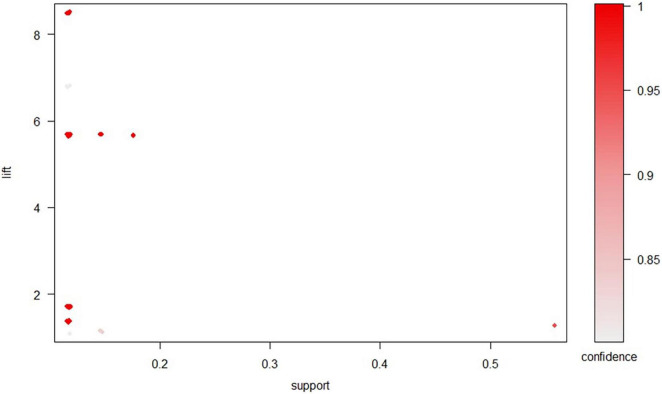
Scatter plot for 61 association rules.

Sixty-one association rules were investigated based on integrated SA location data. The association rules are visually presented based on a scatter plot in Figure 4. The lift of a rule is the ratio of the observed support to that expected if X and Y were independent. The *y*-axis of the scatter plot shows the lift. The results show that all rules had a high lift. The most interesting rules (sc-optimal rules) resided on the support/confidence border (Bayardo and Agrawal, 1999).

The association rules between different individual SA locations were ordered by support. The top 10 *Apriori* algorithm-based association rules of the SA locations are listed in Table 2. The results reveal two groups of interactively selected association rules (1) SAPL_GV20 and SAPL_GV24 (No. 2 and 3 rules); and (2) SAPL_GV20, SAPL_GV24, and ISSA_MS6_i (No. 7, 8, and 9 rules). The results also show that [ISSA_MS7_i] ≥ [ISSA_MS6_i] is the rule with highest support.

**TABLE 2:** Top 10 *Apriori* algorithm-based association rules of the scalp acupuncture locations.

No	Association rules	Support	Confidence	Lift
1	[ISSA_MS7_i] ⇒ [ISSA_MS6_i]	0.5714286	0.9523810	1.282051
2	[SAPL_GV24] ⇒ [SAPL_GV20]	0.1714286	1.0000000	5.833333
3	[SAPL_GV20] ⇒ [SAPL_GV24]	0.1714286	1.0000000	5.833333
4	[SAPL_GV24] ⇒ [ISSA_MS6_i]	0.1428571	0.8333333	1.121795
5	[SAPL_GV20] ⇒ [ISSA_MS6_i]	0.1428571	0.8333333	1.121795
6	[ISSA_MS14] ⇒ [ISSA_MS6_i]	0.1428571	0.8333333	1.121795
7	[SAPL_GV20, SAPL_GV24] ⇒ [ISSA_MS6_i]	0.1428571	0.8333333	1.121795
8	[ISSA_MS6_i, SAPL_GV24] ⇒ [SAPL_GV20]	0.1428571	1.0000000	5.833333
9	[ISSA_MS6_i, SAPL_GV20] ⇒ [SAPL_GV24]	0.1428571	1.0000000	5.833333
10	[SAPL _Ex.HN3] ⇒ [SAPL_GV24]	0.1142857	1.0000000	5.833333

**FIGURE 5:**
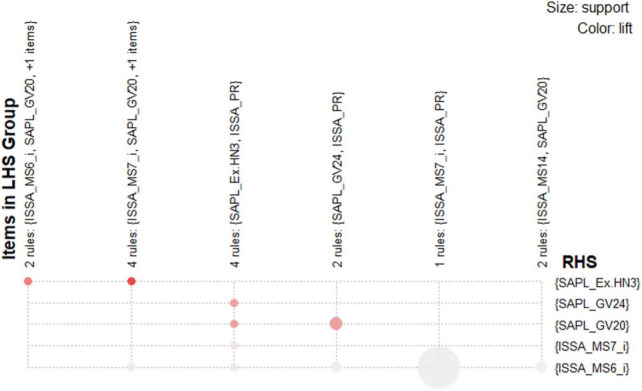
Grouped matrix for 15 association rules.

With respect to the grouped matrix of the association rules, we used graph-based visualization by color or size, as shown in Figure 5. This plot offered an apparent representation of association rules and was appropriate for very small sets of rules to avoid a cluttered presentation. The sets of items (for short item sets) X and Y are called antecedent (left-hand-side or LHS) and consequent (right-hand-side or RHS) of the rule. Often, rules are restricted to only a single item in the consequent. According to the R package “arulesViz”, (Hahsler, 2017) the results of the grouped matrix for 10 rules reveal that the highest interest group (top-left hand corner) consists of two rules, which contain ISSA_MS6_i and SAPL_GV20, and an additional item in the antecedent; moreover, all rules have the consequent SAPL_Ex.HN3. The results also show four rules, including ISSA_MS7_i and SAPL_GV20 with consequent SAPL _Ex.HN3; contain SAPL_Ex.HN3 and ISSA_PR with SAPL_GV24 or SAPL_GV20.

### Quality assessment of the included studies

The quality assessment results are presented in Supplementary Table 4. Of the 35 included RCTs, five RCTs were determined to have “Good” quality, and the other 30 RCTs have “Fair” quality. There are several reasons that affected the quality of the included articles: (1) most of the articles described an unclear randomization method; (2) most of the articles did not report sample size estimation; (3) none of the included studies described allocation concealment; and (4) the acupuncture treatment is difficult to blind the treatment providers and participants.

## Discussion

**FIGURE 6:**
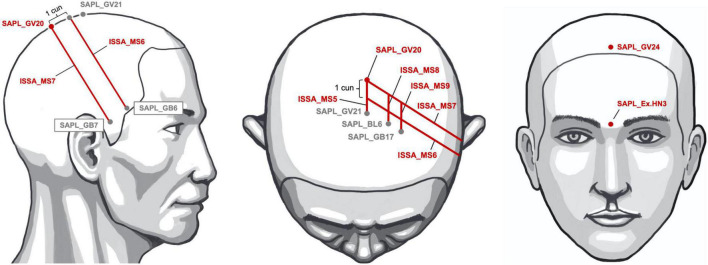
The core scalp acupuncture (SA) location combinations for the treatment of post-stroke hemiparesis. The core scalp acupuncture location combinations [SAPL_GV20 (Baihui), SAPL_GV24 (Shenting), SAPL_Ex.HN3 (Yintang), ISSA_MS6, ISSA_MS7, ISSA_PR (ISSA Parietal region, comprised of ISSA_MS5, ISSA_MS6, ISSA_MS7, ISSA_MS8, and ISSA_MS9)] are shown in red. The other acupoints used as anchor points are shown in gray.

The analysis results indicated that SAPL_GV20, SAPL_GV24, ISSA_MS6_i, ISSA_MS7_i, ISSA_PR, and SAPL_Ex.HN3 can be considered the core SA location combination for the treatment of post-stroke hemiparesis (Figure 6). This study demonstrated evidence-based strategies of SA location selections for post-stroke hemiparesis management and further studies.

In practice, contralateral acupuncture is often used as a treatment in post-stroke rehabilitation for hemiplegic patients. Two functional magnetic resonance imaging (fMRI) studies on unilateral chronic shoulder pain compared brain activation in acupuncture on ipsilateral and contralateral sides and found that treatment on either side alleviates pain intensity and improves shoulder function (Zhang et al., 2018; Yan et al., 2020). A meta-analysis also suggested that there is limited evidence for contralateral acupuncture being superior to ipsilateral acupuncture in post-stroke rehabilitation (Kim et al., 2010). In our findings, ipsilateral ISSA_MS6 and ISSA_MS7 were suggested to be chosen for post-stroke hemiparesis. Future rigorous studies are required to confirm our limited findings.

Liu et al. (2020) reported that SA (ISSA_MS5, MS6, and MS7) enhances functional connections, especially between visual, cognitive, motor control, and planning-related brain regions in acute ischemic stroke patients, by using fMRI examination. Liu H. et al. (2021) also reported that SA (ISSA_MS5, MS6, and MS7) could strengthen the functional activities related to sensory integration, language processing, and motor coordination of the dominant cerebral hemisphere and the motor control bilateral frontal lobe by using the fMRI examination. This present study may provide a scientific basis that SA can modulate the functional connections of brain regions related to the recovery of voluntary movement after brain damage.

According to our result, ISSA_MS6 and ISSA_MS7 are the top two frequently selected SA locations in the included studies. The association rule analysis results also demonstrated that ISSA_MS6_i and ISSA_MS7_i could be considered a part of the core SA location combinations. SA at ISSA_MS6 and ISSA_MS7 was found to reduce plasma endothelin production in patients with stroke (Zhang and Zhang, 2002). Endothelin has been reported to play an essential role in regulating blood–brain barrier (BBB) function in the brain. At the early stage of stroke, TNF-α, IL-1β, and TGF-β are produced by blood-derived inflammatory cells, followed by increasing endothelin production (Koyama, 2013). A previous study showed the protective effects of endothelin type A receptor antagonist on brain edema and injury in a rat model (Matsuo et al., 2001). A recent study also mentioned that bosentan, an antagonist of endothelin type B receptor, could recover the BBB disruption and cerebral edema by downregulating the increasing expression of endothelin-1 and endothelin type B receptors in a mouse model of traumatic brain injury (TBI) (Michinaga et al., 2020). In a recent study, SA on ISSA_MS6 and MS7 decreases neurological impairment and improves escape latency and the target quadrant residence time (Tang et al., 2022). The relative expressions of PI3K and Akt mRNA and protein were also increased in a cerebral ischemia rat model that received SA in this study.

Various studies also showed that SA on ISSA_MS6 reduces β-endorphin of lesioned brain tissue to cure the neuron function, reduces Thromboxane B2 level, increases 6-keto-prostaglandin F1α (6-keto-PGF1α), and maintains blood circulation (Xing et al., 1994; Qin, 2005; He, 2020). The β-endorphin, an endogenous opioid peptide, has various physiological and pathological effects on the human body. The β-endorphin reduces the stress response and the levels of inflammatory cytokines while increasing anti-inflammatory cytokines to minimize the damage to neuronal cells (Pilozzi et al., 2020). A physiological study showed that the nerve cell membranes are depolarized, releasing a large amount of β-endorphin that will occur during cerebral ischemia. The increase in β-endorphin can inhibit ATP metabolism, reduce cAMP production, and change the permeability of cerebral blood vessels. These changes lead to angiogenic edema and serious lesion (Lu et al., 1994). These studies suggest that SA can prevent continued thrombosis and increase vasodilation of neurovasculature in the brain.

Our results demonstrated that SAPL_GV20 (Baihui) and SAPL_GV24 (Shenting) could be considered a part of the core SA location combinations. SA on SAPL_GV20 (Baihui) showed its ability to ease ischemic brain injury by decreasing reactive oxygen species (ROS) and pro-inflammatory cytokines production, such as TNF-α and IL-1β, and by increasing the production of the antioxidant enzyme superoxide dismutase (SOD), neurotrophic factor brain-derived neurotrophic factor (BDNF), and astrocyte activities in a cerebral ischemia rat model (Li et al., 2020b). After treatment with SA on SAPL_GV20 (Baihui), upregulation of cIAP1 expression and inhibition of caspase-8/-9/-3 pathway activation in the ischemic penumbra area were also observed in the ischemic rat brain (Tang et al., 2021). Liu P. et al. (2021) demonstrated that acupuncture on SAPL_GV20 (Baihui) might enhance mitophagy and reduce brain cell apoptosis to ease intracerebral hemorrhage brain damage and improve neurological function in an intracerebral hemorrhage rat model. The previous clinical study investigated whether increased plasma β-endorphin in patients with hemiparesis will be reduced by SA [Baihui (SAPL_GV20) to Qianding (SAPL_GV21), and Shuaigu (SAPL_GB8) point to Xuanli (SAPL_GB6)] (Wu et al., 2000). These lines of evidence suggest that SA might induce neuroprotection mechanisms modulated by various signaling pathways.

Yan et al. (2020) reported that electroacupuncture at SAPL_GV20 (Baihui) and SAPL_GV24 (Shenting) acupoints could improve the learning and memory impairment in a rat model of cerebral ischemia-reperfusion injury and the mechanism may be related to enhancing SOD activity and reducing the accumulation of malondialdehyde (MDA) in the brain tissue (Xiao et al., 2022). A similar study showed that electroacupuncture at SAPL_GV20 and SAPL_GV24 can alleviate neurological deficit and improve cognitive function in cerebral ischemia-reperfusion rats, which may be related to its effects in up-regulating endogenous melatonin levels, inhibiting the activation of astrocytes and protecting damaged neurons in the hippocampus (Zhong et al., 2022). A previous study demonstrated that melatonin might accelerate the process of motor axons’ repair *via* Melatonin 1 (MT1) receptor (Stazi et al., 2021). These studies demonstrate evidence-based that SA might involve in neurogenesis after ischemia-reperfusion injury.

## Limitation of the study

Although we conducted a comprehensive systematic review and analysis, the present study had a few limitations. First, the acupuncture details may affect the treatment effects, such as depth of needling, stimulation method of acupuncture, time of retaining the needle, treatment frequency, and treatment course. However, these factors were not analyzed in this analysis. Second, because many of the SA treatment types did not report the effects in RCT studies, the SA selections in clinical practice may be neglected. Further basic and clinical studies are necessary for a thorough evaluation of combinations of SA locations for the treatment of post-stroke hemiparesis.

## Conclusion

The SAPL_GV20 (Baihui), SAPL_GV24 (Shenting), ISSA_MS6_i (ISSA Anterior Oblique Line of Vertex-Temporal, lesion-ipsilateral), ISSA_MS7_i (ISSA Posterior Oblique Line of Vertex-Temporal, lesion-ipsilateral), ISSA_PR (ISSA Parietal region, comprised of ISSA_MS5, ISSA_MS6, ISSA_MS7, ISSA_MS8, and ISSA_MS9), and SAPL_Ex.HN3 (Yintang) can be considered the core SA location combinations for the treatment of post-stroke hemiparesis (Figure 6). We recommend the core SA combinations for further animal studies, clinical trials, and treatment strategies.

## Data availability statement

The original contributions presented in this study are included in the article/Supplementary material, further inquiries can be directed to the corresponding authors.

## Author contributions

Y-FW, W-YC, Y-YS, and P-CH: study conception and design. Y-FW and W-YC: data collection. G-TL, C-YK, and P-CH: statistical analysis. Y-FW, W-YC, C-TL, Y-YS, C-CL, M-LC, and P-CH: interpretation of results. Y-FW, M-LC, and P-CH: drafting manuscript. C-TL: visualization. M-LC and P-CH: project administration. All authors reviewed the results and approved the final version of the manuscript.

## Glossary

ADL, activities of daily living; BBB, blood–brain barrier; BDNF, brain-derived neurotrophic factor; FMA, Fugl-Myer Assessment; fMRI, Functional magnetic resonance imaging; HRQL, health-related quality of life; IL-1β, Interleukin-1β; ISSA, International Standard Scalp Acupuncture; ISSA_FR, forehead region; ISSA_MS1, Middle Line of forehead; ISSA_MS2, Lateral Line 1 of forehead; ISSA_MS3, Lateral Line 2 of forehead; ISSA_MS4, Lateral Line 3 of Forehead; ISSA_MS5, Middle Line Vertex; ISSA_MS6 M2/5, Motor Area (MTA) Middle 2/5; ISSA_MS6_b, Anterior Oblique Line of Vertex-Temporal_Both side; ISSA_MS6_c, Anterior Oblique Line of Vertex-Temporal_Lesion-contralateral; ISSA_MS6_i, Anterior Oblique Line of Vertex-Temporal_Lesion-ipsilateral; ISSA_MS7_i, Posterior Oblique Line of Vertex-Temporal_Lesion-ipsilateral; ISSA_MS8, Lateral Line 1 of Vertex; ISSA_MS9, Lateral Line 2 of Vertex; ISSA_MS10_i, Anterior Temporal Line-ipsilateral; ISSA_MS11_i, Posterior Temporal Line-ipsilateral; ISSA_MS12, Upper-Middle Line of Occiput; ISSA_MS13, Upper-Lateral Line of Occiput; ISSA_MS14, Lower-Lateral Line of Occiput; ISSA_OR, occipital region; ISSA_PR, parietal region; ISSA_TR, temporal region; Jiao, Jiao Sun-Fa Scalp Acupuncture Style; Jiao_B, Balance area; Jiao_VM, voluntary movement area; LHS, left-hand-side; Lin, Lin Xue-Jian Scalp Acupuncture Style; Lin_FFN, Frontal five-needle; Lin_PM, premotor area; Lin_TTN, temporal three-needle; MDA, malondialdehyde; MT1, melatonin 1; RCT, randomized-controlled trial; RHS, right-hand-side; ROS, reactive oxygen species; SA, scalp acupuncture; SAPL, WHO Standard Acupuncture Point Locations; SAPL _Ex-HN1, Sishencong; SAPL _Ex-HN3, Yintang; SAPL _GB20, Fengchi; SAPL _GV20, Baihui; SAPL _GV20-Ex-HN5, GV20-Ex-HN5 Line; SAPL _GV21, Qianding; SAPL _GV23, Shangxing; SAPL_ GV24, Shenting; SAPL _GV26, Renzhong (Shuigou); SOD, superoxide dismutase; TBI, traumatic brain injury; TGF-β, transforming growth factor-β; TNF-α, tumor necrosis factor-α; Zhu, Zhu Ming-Qing Scalp Acupuncture Style; Zhu_C, cervical area; Zhu_LJ, lower Jiao area; Zhu_UL_i, upper limb area_Lesion-ipsilateral; Zhu_VPF, vertex-perineum-foot area.
