# Supplementary material for: Combinations of scalp acupuncture location for the treatment of post-stroke hemiparesis: A systematic review and Apriori algorithm-based association rule analysis
Source: Front Neurosci. 2022 Aug 5;16:956854. doi: 10.3389/fnins.2022.956854 (PMC9389219; doi:10.3389/fnins.2022.956854)
Supplement: Supplementary file 1 [file Data_Sheet_1.pdf]

Supplementary Materials

Combinations of scalp acupuncture location for the treatment of post-stroke hemiparesis: a systematic review and Apriori algorithm-based association rule analysis

Yu-Fang Wang, Wei-Yi Chen, Yi-Ying Shen, Chou-Chin Lan, Guan-Ting Liu, Chan-Yen Kuo, Mao-Liang Chen, Po-Chun Hsieh

Supplementary Table 1. Search strategy and results	1
Supplementary Table 2. Binary data of the scalp acupuncture locations	2
Supplementary Table 3. List of excluded articles with reasons after full-text evaluation	3
Supplementary Table 4. Quality assessment of the retrieved studies	10
PRISMA 2020 Checklist	12

Supplementary Table 1. Search strategy and results

Search strategy and results in PubMed		
Search	Query	Results
#1	stroke	399289
#2	scalp acupuncture	424
#3	#1 AND #2	128
**Search strategy and results in Embase**		
Search	Query	Results
#1	stroke	618563
#2	scalp acupuncture	430
#3	#1 AND #2	117
**Search strategy and results in Cochrane Library**		
Search	Query	Results
#1	stroke	75630
#2	scalp acupuncture	446
#3	#1 AND #2	198
**Search strategy and results in Airiti Library**		
Search	Query	Results
#1	中風 OR 卒中	8055
#2	頭針 OR 頭皮針	6019
#3	#1 AND #2	104
**Search strategy and results in China National Knowledge Infrastructure (CNKI)**		
Search	Query	Results
#1	中風 + 卒中	112062
#2	頭針 + 頭皮針	3096
#3	#1 * #2	768

Supplementary Table 2. Binary data of the scalp acupuncture locations

Study	Xie 2007	Li 2009	Ma 2010	Fu 2011	Qin 2013	Kong 2014	Qin D 2015	Tan 2015	Xu 2015	Zhang 2015	Chen 2016	Dou 2016	Liu 2016	Pan 2017	Wang 2017	Yang 2017	Yin YH 2017	Hu HY 2018	Xiao L 2018	Xu 2018	Qi 2018	Chen 2019	Hu 2019	Li 2019	Ma 2019	Sun XQ 2019	Ye 2019	Zhang XY 2019	Zhang HL 2019	Zhao 2019	Zhu 2019	Xiong 2020	Yin 2020	Wang 2020	Zhang 2021
ISSA_PR	0	0	0	0	0	0	0	0	0	0	0	0	0	0	0	1	0	0	0	0	0	0	0	0	0	0	0	0	0	0	0	0	0	0	0
ISSA_OR	0	0	0	0	0	1	0	0	0	0	0	0	0	0	0	0	0	0	0	0	0	0	0	0	0	0	0	0	0	0	0	0	0	0	0
ISSA_MS1	1	0	0	1	0	0	1	0	0	0	0	0	0	0	0	0	0	0	0	0	0	0	0	0	0	0	0	0	0	0	0	0	0	0	0
ISSA_MS5	1	1	0	0	0	0	1	1	0	0	0	0	0	0	0	0	0	0	0	1	0	1	1	1	1	0	0	0	1	0	0	0	0	0	0
ISSA_MS6_i	0	1	0	1	0	1	1	1	1	1	1	1	1	0	1	0	1	1	1	1	1	1	1	0	1	1	1	1	0	1	1	0	1	0	1
ISSA_MS6_c	0	0	1	0	0	0	0	0	0	0	0	0	0	0	0	0	0	0	0	0	0	0	0	0	0	0	0	0	0	0	0	0	0	0	0
ISSA_MS6_b	0	0	0	0	0	0	0	0	0	0	0	0	0	0	0	0	0	0	0	0	0	0	0	0	0	0	0	0	0	0	0	0	0	1	0
ISSA_MS6_M2/5	0	0	0	0	0	0	0	0	0	1	0	0	0	0	1	0	0	0	0	0	0	0	0	0	0	0	0	0	0	0	0	0	0	0	0
ISSA_MS7_i	0	1	0	1	0	1	1	0	1	0	1	1	0	0	1	0	1	1	0	0	1	0	1	1	1	1	1	1	0	1	1	0	1	0	1
ISSA_MS8	0	0	0	0	0	1	0	0	0	0	0	0	0	0	0	0	0	0	0	1	0	1	0	1	0	0	0	0	0	0	0	0	0	0	0
ISSA_MS9	0	0	0	0	0	1	0	0	0	0	0	0	0	0	0	0	0	0	0	0	0	0	0	0	0	0	0	0	0	1	0	0	0	0	0
ISSA_MS10_i	1	0	1	0	0	1	0	0	0	0	0	0	0	0	0	0	0	0	0	0	0	0	0	0	0	0	0	0	0	0	0	0	0	0	0
ISSA_MS11_i	1	0	0	0	0	1	0	0	0	0	0	0	0	0	0	0	0	0	0	0	0	0	0	0	0	0	0	0	0	0	0	0	0	0	0
ISSA_MS13	0	0	0	0	0	1	0	0	0	0	0	0	0	0	0	0	0	0	0	0	0	0	0	0	0	0	0	0	0	0	0	0	0	0	0
ISSA_MS14	0	0	0	0	0	1	0	0	0	0	1	0	0	0	1	0	0	0	0	1	0	1	0	0	0	0	0	0	1	0	0	0	0	0	0
SAPL_GB20	0	0	0	0	0	0	0	0	0	0	0	0	0	0	0	0	0	0	0	0	0	0	0	0	0	0	0	0	0	0	0	1	0	0	0
SAPL_GV20	0	0	0	0	0	0	0	0	1	1	0	1	0	0	0	0	0	1	0	0	0	0	0	0	0	0	1	0	0	0	0	1	0	0	0
SAPL_GV21	0	0	0	0	0	0	0	0	0	0	0	0	0	0	0	1	0	0	0	0	0	0	0	0	0	0	0	0	0	0	0	0	0	0	0
SAPL_GV23	0	0	0	0	0	0	0	0	0	0	0	0	0	0	0	0	0	0	0	0	0	0	0	0	0	0	1	0	0	0	0	0	0	0	0
SAPL_GV24	0	0	0	0	0	0	0	0	1	1	0	1	0	0	0	0	0	1	0	0	0	0	0	0	0	0	1	0	0	0	0	1	0	0	0
SAPL_GV26	0	0	0	0	0	0	0	0	0	0	0	0	0	0	0	0	0	0	0	0	0	1	0	0	0	0	0	0	0	0	0	0	0	0	0
SAPL_Ex-HN1	0	0	0	0	0	0	0	0	0	1	0	0	0	1	1	0	0	0	0	0	0	0	0	0	0	0	0	0	0	0	0	1	0	0	0
SAPL_Ex-HN3	0	0	0	0	0	0	0	0	1	0	0	1	0	0	0	0	0	1	0	0	0	0	0	0	0	0	1	0	0	0	0	0	0	0	0
SAPL_GV20-Ex-HN5	0	0	0	0	1	0	0	0	0	0	0	0	0	0	0	0	0	0	0	0	0	0	0	0	0	0	0	0	0	0	1	0	0	0	0
Zhu_C	0	0	0	0	0	1	0	0	0	0	0	0	0	0	0	0	0	0	0	0	0	0	0	0	0	0	0	0	0	0	0	0	0	0	0
Zhu_UL	0	0	0	0	0	0	0	0	0	0	0	0	0	1	0	0	0	0	0	0	0	0	0	0	0	0	0	0	0	0	0	0	0	0	0
Zhu_VPF	0	0	0	0	0	0	0	0	0	0	0	0	0	1	0	0	0	0	0	0	0	0	0	0	0	0	0	0	0	0	0	0	0	0	0
Zhu_LJ	0	0	0	0	0	0	0	0	0	0	0	0	0	1	0	0	0	0	0	0	0	0	0	0	0	0	0	0	0	0	0	0	0	0	0
Jiao_B	0	0	0	0	0	0	0	0	0	0	0	0	0	0	1	0	0	0	0	0	0	0	0	0	0	0	0	0	0	0	0	0	0	0	0
Jiao_VM	0	0	0	0	0	0	0	0	0	0	0	0	0	0	1	0	0	0	0	0	0	0	0	0	0	0	0	0	0	0	0	0	0	0	0
Lin_PM	0	0	0	0	0	0	0	0	0	0	0	0	0	0	1	0	0	0	0	0	0	0	0	0	0	0	0	0	0	0	0	0	0	0	0
Lin_TTN	0	0	0	0	0	0	0	0	0	0	0	0	0	0	1	0	0	0	0	0	0	0	0	0	0	0	0	0	0	0	0	0	0	0	0
Lin_FFN	0	0	0	0	0	0	0	0	0	0	0	0	0	0	1	0	0	0	0	0	0	0	0	0	0	0	0	0	0	0	0	0	0	0	0

Supplementary Table 3. List of excluded articles with reasons after full-text evaluation

Not RCT (n=28)	
#	Title
1	Liu H, Jiang Y, Wang N, Yan H, Chen L, Gao J, Zhang J, Qu S, Liu S, Liu G, Huang Y, Chen J. Scalp acupuncture enhances local brain regions functional activities and functional connections between cerebral hemispheres in acute ischemic stroke patients. Anat Rec (Hoboken). 2021 Nov;304(11):2538-2551. doi: 10.1002/ar.24746.
2	Hu X, Li B, Wang X. Scalp acupuncture therapy combined with exercise can improve the ability of stroke patients to participate in daily activities. Complement Ther Clin Pract. 2021 May;43:101343. doi: 10.1016/j.ctcp.2021.101343.
3	Zhao N, Zhang H, Liu T, Liu J, Xiang Y, Shu G, Li C, Xie J, Chen L. Neuromodulatory Effect of Sensorimotor Network Functional Connectivity of Temporal Three-Needle Therapy for Ischemic Stroke Patients with Motor Dysfunction: Study Protocol for a Randomized, Patient-Assessor Blind, Controlled, Neuroimaging Trial. Evid Based Complement Alternat Med. 2021 Jan 4;2021:8820324. doi: 10.1155/2021/8820324.
4	Xu J, Pei J, Fu QH, Wang LY, Zhan YJ, Tao L. Earlier Acupuncture Enhancing Long-Term Effects on Motor Dysfunction in Acute Ischemic Stroke: Retrospective Cohort Study. Am J Chin Med. 2020;48(8):1787-1802. doi: 10.1142/S0192415X20500895.
5	Sun L, Fan Y, Fan W, Sun J, Ai X, Qiao H. Efficacy and safety of scalp acupuncture in improving neurological dysfunction after ischemic stroke: A protocol for systematic review and meta-analysis. Medicine (Baltimore). 2020 Aug 21;99(34):e21783. doi: 10.1097/MD.0000000000021783.
6	Xu J, Pei J, Fu QH, Zhan YJ. The Prognostic Value of Traditional Chinese Medicine Symptoms in Acute Ischemic Stroke: A Pilot Study. Evid Based Complement Alternat Med. 2020 Jul 15;2020:1520851. doi: 10.1155/2020/1520851.
7	Wang X, Zhang Q, Cui B, Huang L, Wang D, Ye L, Sun J. Scalp-cluster acupuncture with electrical stimulation can improve motor and living ability in convalescent patients with post-stroke hemiplegia. J Tradit Chin Med. 2018 Jun;38(3):452-456.
8	Tian L, Du X, Wang J, Sun R, Zhang Z, Yuan B, Zhang X, Li X, Zhang T. [Comparative study on the effects between manual acupuncture and electroacupuncture for hemiplegia after acute ischemic stroke]. Zhongguo Zhen Jiu. 2016 Nov 12;36(11):1121-1125. Chinese. doi: 10.13703/j.0255-2930.2016.11.001.
9	You YN, Cho MR, Kim JH, Park JH, Park GC, Song MY, Choi JB, Han JY. Assessing the quality of reports about randomized controlled trials of scalp acupuncture combined with another treatment for stroke. BMC Complement Altern Med. 2017 Sep 6;17(1):452. doi: 10.1186/s12906-017-1950-6.
10	Wang J, Pei J, Khiati D, Fu Q, Cui X, Song Y, Yan M, Shi L, Cai Y, Ma Y. Acupuncture treatment on the motor area of the scalp for motor dysfunction in patients with ischemic stroke: study protocol for a randomized controlled trial. Trials. 2017 Jun 20;18(1):287. doi: 10.1186/s13063-017-2000-x.
11	Zhang LF, Wang LY, Li LY, Zeng XQ, He Y, Dai HS. [Effect of Scalp Electroacupuncture Combined Constraint-induced Movement Therapy on Move- ment Function of Ischemic Stroke Patients' Upper Limbs]. Zhongguo Zhong Xi Yi Jie He Za Zhi. 2017 Mar;37(3):314-318.

12	Han JY, Kim JH, Park JH, Song MY, Song MK, Kim DJ, You YN, Park GC, Choi JB, Cho MR, Shin JC, Cho JH. Scalp acupuncture and electromagnetic convergence stimulation for patients with cerebral infarction: study protocol for a randomized controlled trial. Trials. 2016 Oct 11;17(1):490. doi: 10.1186/s13063-016-1611-y.
13	Du XZ, Bao CL, Dong GR, Yang XM. Immediate effects of scalp acupuncture with twirling reinforcing manipulation on hemiplegia following acute ischemic stroke: a hidden association study. Neural Regen Res. 2016 May;11(5):758-64. doi: 10.4103/1673-5374.182702.
14	Lang Y, Cui FY, Li KS, Tan ZJ, Zou YH. [Imaging Observation of Scalp Acupuncture on Brain Gray Matter Injury in Stroke Patients with Cerebral Infarction]. Zhongguo Zhong Xi Yi Jie He Za Zhi. 2016 Mar;36(3):294-9.
15	Chen L, Fang J, Ma R, Froym R, Gu X, Li J, Chen L, Xu S, Ji C. Acupuncture for acute stroke: study protocol for a multicenter, randomized, controlled trial. Trials. 2014 Jun 8;15:214. doi: 10.1186/1745-6215-15-214.
16	Wong YM. Scalp electrical acupuncture. J Altern Complement Med. 2013 Jul;19(7):677. doi: 10.1089/acm.2012.0832.
17	Santos AB, Gozzani JL. Acupuncture as adjuvant therapy in thalamic syndrome: case report. Rev Bras Anestesiol. 2011 Jan-Feb;61(1):88-94. doi: 10.1016/S0034-7094(11)70010-1.
18	Bao F, Wang DH, Zhang YX, Wang FQ, Sun H. [Comparison of therapeutic effects between body acupuncture and scalp acupuncture combined with body acupuncture on atherosclerotic cerebral infarction at acute stage]. Zhongguo Zhen Jiu. 2008 Jan;28(1):10-2.
19	Yamamoto T, Schockert T, Boroojerdi B. Treatment of juvenile stroke using Yamamoto New Scalp Acupuncture (YNSA) - a case report. Acupunct Med. 2007 Dec;25(4):200-2. doi: 10.1136/aim.25.4.200.
20	Ouyang G, Jia SW, Wang F, Shi Y, Gao Z. [Effects of electroacupuncture of different frequencies on cerebral blood perfusion and cerebral function in the patient of stroke]. Zhongguo Zhen Jiu. 2005 Nov;25(11):776-8.
21	Zhao DG, Mu JP. [Clinical study on scalp acupuncture combined with sports therapy for rehabilitation of poststroke hemiplegia]. Zhongguo Zhen Jiu. 2005 Jan;25(1):19-20.
22	Cui H, Zhang HF, Ren ZM, Yu ZS, Tang Q. [Observation on therapeutic effect of pulse magnetic acupuncture at scalp acupoints on acute cerebral infarction]. Zhongguo Zhen Jiu. 2005 Aug;25(8):526-8.
23	Inoue I, Chen L, Zhou L, Zeng X, Wang H. Reproduction of scalp acupuncture therapy on strokes in the model rats, spontaneous hypertensive rats-stroke prone (SHR-SP). Neurosci Lett. 2002 Nov 29;333(3):191-4. doi:10.1016/s0304-3940(02)01032-7.
24	Zhou J, Zhang F. A research on scalp acupuncture for cerebral infarction. J Tradit Chin Med. 1997 Sep;17(3):194-7.
25	Hu J. Acupuncture treatment of wind stroke. J Tradit Chin Med. 1996 Dec;16(4):307-11.
26	Shi Y, Song Z. Clinical observation on head acupuncture treatment of 100 cases of hemiplegia with the through-to-through chou-tian technique. J Tradit Chin Med. 1996 Jun;16(2):129-33.
27	Wang Y, Xu G, Li G, Li D, Fang Y, Li Y, Wu F. Treatment of apoplectic hemiplegia with scalp

	acupuncture in relation to CT findings. J Tradit Chin Med. 1993 Sep;13(3):182-4.
28	Xiao J. [Clinical observation of 50 cases of hemiplegia treated by acupuncture]. Zhen Ci Yan Jiu. 1993;18(3):172-3.
**Review article (n=18)**	
#	Title
1	Huang YJ, Huang CS, Leng KF, Sung JY, Cheng SW. Efficacy of Scalp Acupuncture in Patients With Post-stroke Hemiparesis: Meta-Analysis of Randomized Controlled Trials. Front Neurol. 2021 Dec 9;12:746567. doi: 10.3389/fneur.2021.746567.
2	Hang X, Li J, Zhang Y, Li Z, Zhang Y, Ye X, Tang Q, Sun W. Efficacy of frequently-used acupuncture methods for specific parts and conventional pharmaceutical interventions in treating post-stroke depression patients: A network meta-analysis. Complement Ther Clin Pract. 2021 Nov;45:101471. doi:10.1016/j.ctcp.2021.101471.
3	Belskaya GN, Stepanova SB, Makarova LD, Sergienko DA, Krylova LG, Antimonova KV. Akupunktura v profilaktike i lechenii insul'ta: obzor zarubezhnykh issledovanii [Acupuncture in the prevention and treatment of stroke: a review of foreign studies]. Vopr Kurortol Fizioter Lech Fiz Kult. 2020;97(2):68-77. Russian. doi: 10.17116/kurort20209702168.
4	Tang HY, Tang W, Yang F, Wu WW, Shen GM. Efficacy of acupuncture in the management of post-apoplectic aphasia: a systematic review and meta-analysis of randomized controlled trials. BMC Complement Altern Med. 2019 Oct 25;19(1):282. doi: 10.1186/s12906-019-2687-1.
5	Young-Nim Y, Gwang-Cheon P, Myung-Rae C, Min-Yeong S, Chang-Su N, Jae-Young H, Jae-Hong K. Meta-analysis on randomized controlled trials for scalp acupuncture treatment of stroke: A systematic review. J Tradit Chin Med. 2018 Aug;38(4):465-479. PMID: 32186072.
6	Tian L, Wang JH, Sun RJ, Zhang XH, Yuan B, Du XZ. [Development of Researches on Scalp Acupuncture for Ischemic Stroke]. Zhen Ci Yan Jiu. 2016 Feb;41(1):87-9, 93.
7	Li HQ, Li JH, Liu AJ, Ye MY, Zheng GQ. GV20-based acupuncture for animal models of acute intracerebral haemorrhage: a preclinical systematic review and meta-analysis. Acupunct Med. 2014 Dec;32(6):495-502. doi: 10.1136/acupmed-2014-010546. Epub 2014 Oct 23.
8	Chen LF, Fang JQ, Chen LN, Wang C. [Achievements and enlightenment of modern acupuncture therapy for stroke based on the neuroanatomy]. Zhen Ci Yan Jiu. 2014 Apr;39(2):164-8.
9	Wang WW, Xie CL, Lu L, Zheng GQ. A systematic review and meta-analysis of Baihui (GV20)-based scalp acupuncture in experimental ischemic stroke. Sci Rep. 2014 Feb 5;4:3981. doi: 10.1038/srep03981.
10	Sun Y, Xue SA, Zuo Z. Acupuncture therapy on apoplectic aphasia rehabilitation. J Tradit Chin Med. 2012 Sep;32(3):314-21. doi: 10.1016/s0254-6272(13)60031-x.
11	Wang Y, Shen J, Wang XM, Fu DL, Chen CY, Lu LY, Lu L, Xie CL, Fang JQ, Zheng GQ. Scalp acupuncture for acute ischemic stroke: a meta-analysis of randomized controlled trials. Evid Based Complement Alternat Med. 2012;2012:480950. doi: 10.1155/2012/480950.
12	Hao JJ, Hao LL. Review of clinical applications of scalp acupuncture for paralysis: an excerpt from chinese scalp acupuncture. Glob Adv Health Med. 2012 Mar;1(1):102-21. doi: 10.7453/gahmj.2012.1.1.017.

13	Wang HQ, Wang F, Liu JH, Dong GR. [Introduction on the schools of the scalp acupuncture for treatment of the stroke hemiplegia]. Zhongguo Zhen Jiu. 2010 Sep;30(9):783-6.
14	Zhang S, Li N, Liu M. Use of acupuncture for stroke in China. Acupunct Med. 2009 Dec;27(4):146. doi: 10.1136/aim.2009.001669.
15	Zheng GQ. Methodological standards for experimental research on stroke using scalp acupuncture. Acupunct Electrother Res. 2009;34(1-2):1-13. doi: 10.3727/036012909803861086.
16	Liang W, Zhu BC. [Chief physician SHAN Yong-Hua's clinical experiences]. Zhongguo Zhen Jiu. 2008 Feb;28(2):129-32.
17	Ouyang Q, Zhou W, Zhang CM. [The key of increasing the therapeutic effect of scalp acupuncture on hemiplegia due to stroke]. Zhongguo Zhen Jiu. 2007 Oct;27(10):773-6.
18	Liu LG, Zhang HX. [Significance of nerve stem cells in the research on clinical treatment of ischemic stroke with scalp-acupuncture therapy]. Zhen Ci Yan Jiu. 2007 Aug;32(4):277-80.
**Without target treatment (n=6)**	
#	Title
1	Zhang Q, Wang Y, Ji G, Cao F, Hu G, Cong D, Xu X, Song B. Standardization of rehabilitation program for post-apoplectic limb spasm treated by Tongjing Tiaoxing tuina and scalp acupuncture with physical therapy. Medicine (Baltimore). 2020 May 22;99(21):e20368. doi: 10.1097/MD.0000000000020368.
2	Litscher G, Zhang X, Sheng Z, Jing XH, Wang L. Multimodal Laser Stimulation and Traditional Needle Acupuncture in Post-Stroke Patients-A Pilot Cross-Over Study with Results from Near Infrared Spectroscopy. Medicines (Basel). 2019 Dec 16;6(4):115. doi: 10.3390/medicines6040115.
3	Chen XQ, Zhu MY, Zou YC, Wu ZH, Peng YX, Huang F. [Effect of "Tongyuan" acupuncture treatment on success rate of extubation in stroke patients undergoing tracheotomy]. Zhen Ci Yan Jiu. 2019 Sept 25;44(9):663-7. Chinese. doi: 10.13702/j.1000-0607.180718.
4	Cheng YH, Lu XY, Yu XG. [Clinical observation on therapeutic effect of three-step acupuncture for the secondary prevention of ischemic cerebral apoplexy]. Zhongguo Zhen Jiu. 2010 Apr;30(4):270-4.
5	Seo J, Lee HS, Ha E, Park HJ, Park HK, Lee H, Kang S, Yin C, Kim J, Leem KH, Kim EH, Ryu Y, Choi S, Chung JH. Efficacy of combined treatment by scalp and penetration acupunctures with TKM medication (tang) on stroke patients. Neurol Res. 2007;29 Suppl 1:S38-41. doi: 10.1179/016164107X172275.
6	Liu Y. Treatment of pseudobulbar paralysis by scalp acupuncture and sublingual needling. J Tradit Chin Med. 2004 Mar;24(1):26-7.
**Without target outcome (n=37)**	
#	Title
1	Xu L, Li F, Wang M, Yan XZ, Du YH. [Scalp acupuncture combined with suspension training for balance dysfunction in patients with stroke: a randomized controlled trial]. Zhongguo Zhen Jiu. 2021 Dec 12;41(12):1308-12. Chinese. doi: 10.13703/j.0255-2930.20201217-0002.
2	Zhang SH, Wang YL, Zhang CX, Zhang CP, Xiao P, Li QF, Liang WR, Pan XH, Zhou MC. Effect of Interactive Dynamic Scalp Acupuncture on Post-Stroke Cognitive Function, Depression, and

	Anxiety: A Multicenter, Randomized, Controlled Trial. Chin J Integr Med. 2022 Feb;28(2):106-115. doi: 10.1007/s11655-021-3338-1.
3	Lou XQ, Liu X, Liu CH, Lin HJ, Liu H, Ling J. [Therapeutic effect of electric-balance stimulation with scalp acupuncture for motor aphasia after cerebral infarction]. Zhongguo Zhen Jiu. 2021 Nov 12;41(11):1211-5. Chinese. doi: 10.13703/j.0255-2930.20210302-k0005.
4	Liu Z, Huang J, Xu Y, Wu J, Tao J, Chen L. Cost-effectiveness of speech and language therapy plus scalp acupuncture versus speech and language therapy alone for community-based patients with Broca's aphasia after stroke: a post hoc analysis of data from a randomised controlled trial. BMJ Open. 2021 Sep 6;11(9):e046609. doi: 10.1136/bmjopen-2020-046609.
5	Zhang CX, Zhang SH, Wang YL, Zhang CP, Li QF, Pan WY, Liang WR. [Interactive scalp acupuncture for cognitive dysfunction after stroke: a randomized controlled trial]. Zhongguo Zhen Jiu. 2021 Mar 12;41(3):252-6. Chinese. doi: 10.13703/j.0255-2930.20200212-k0003.
6	Liu H, Chen L, Zhang G, Jiang Y, Qu S, Liu S, Huang Y, Chen J. Scalp Acupuncture Enhances the Functional Connectivity of Visual and Cognitive-Motor Function Network of Patients with Acute Ischemic Stroke. Evid Based Complement Alternat Med. 2020 Dec 2;2020:8836794. doi: 10.1155/2020/8836794.
7	Jin HP, Li XL, Ye QJ, Wang Y. [Effect of electrical stimulation with bilateral scalp acupuncture on time parameters in video fluoroscopic swallowing study and cortical excitability in patients with dysphagia after cortical stroke]. Zhen Ci Yan Jiu. 2020 Jun 25;45(6):473-9. Chinese. doi: 10.13702/j.1000-0607.190697.
8	Chen J, Li H, Zeng C, Li J, Zhao B. Evaluation of the recovery outcome of poststroke cognitive impairment after cluster needling of scalp acupuncture therapy based on functional near-infrared spectroscopy. Brain Behav. 2020 Aug;10(8):e01731. doi: 10.1002/brb3.1731.
9	Lang Y, Li KS, Yang JY, Cui FY, Bai W, Liang CX. [Effect of acupuncture at the anterior oblique parietotemporal line on gray matter remodeling in patients with hemiplegia of cerebral infarction]. Zhen Ci Yan Jiu. 2020 Feb 25;45(2):141-7. Chinese. doi: 10.13702/j.1000-0607.1907576.
10	Wang HQ, Dong GR, Bao CL, Jiao ZH. Immediate effect of scalp acupuncture on the gait of patients with subacute intracerebral haemorrhage analysed by three- dimensional motion: secondary analysis of a randomised controlled trial. Acupunct Med. 2018 Apr;36(2):71-79. doi: 10.1136/acupmed-2016-011272.
11	Zhao N, Zhang J, Qiu M, Wang C, Xiang Y, Wang H, Xie J, Liu S, Wu J. Scalp acupuncture plus low-frequency rTMS promotes repair of brain white matter tracts in stroke patients: A DTI study. J Integr Neurosci. 2018;17(1):61-69. doi: 10.31083/JIN-170043.
12	Wang JH, Zhao M, Bao YC, Shang JF, Yan Q, Zhang ZC, Du XZ, Jiang H, Zhang WD. [Effect of Scalp-acupuncture Treatment on Levels of Serum High-sensitivity C-reactive Protein, and Pro-inflammatory Cytokines in Patients with Acute Cerebral Infarction]. Zhen Ci Yan Jiu. 2016 Feb;41(1):80-4.
13	Chu JM, Bao YH, Zhu M. [Effects of Acupuncture Intervention Combined with Rehabilitation on Standing-balance-walking Ability in Stroke Patients]. Zhen Ci Yan Jiu. 2015 Dec;40(6):474-8.
14	Jiang Y, Yang Y, Xiang R, Chang E, Zhang Y, Zuo B, Zhang Q. [Clinical study of post-stroke speech

	apraxia treated with scalp electric acupuncture under anatomic orientation and rehabilitation training]. Zhongguo Zhen Jiu. 2015 Jul;35(7):661-4.
15	Chang L, He PL, Zhou ZZ, Li YH. [Efficacy observation of dysphagia after acute stroke treated with acupuncture and functional electric stimulation]. Zhongguo Zhen Jiu. 2014 Aug;34(8):737-40.
16	Yu Z. [Clinical research of catgut implantation at scalp reflecting foci for post-stroke sensory disturbance]. Zhongguo Zhen Jiu. 2014 Jul;34(7):631-5.
17	Guo AS, Li AH, Chen X, Chen WG, Sun L. [Effect of acupoint catgut embedding on motor function and serum high sensitivity C-reactive protein and IL-6 levels in patients with acute cerebral infarction]. Zhen Ci Yan Jiu. 2013 Jun;38(3):224-8, 258.
18	He K, Zhang H, Wu QM, Yan J, Shi ZE, Dai SJ, Li DD. [The combined application of scalp and body acupuncture by stages for low limb dysfunction of patients with apoplexy]. Zhongguo Zhen Jiu. 2012 Oct;32(10):887-90.
19	Hegyi G, Szigeti GP. Rehabilitation of stroke patients using Yamamoto New Scalp Acupuncture: a pilot study. J Altern Complement Med. 2012 Oct;18(10):971-7. doi: 10.1089/acm.2011.0047.
20	Hsing WT, Imamura M, Weaver K, Fregni F, Azevedo Neto RS. Clinical effects of scalp electrical acupuncture in stroke: a sham-controlled randomized clinical trial. J Altern Complement Med. 2012 Apr;18(4):341-6. doi: 10.1089/acm.2011.0131.
21	Hao JJ, Zhongren S, Xian S, Tiansong Y. Chinese scalp acupuncture for cerebral palsy in a child diagnosed with stroke in utero. Glob Adv Health Med. 2012 Mar;1(1):14-7. doi: 10.7453/gahmj.2012.1.1.005.
22	Li H, Hou ZW, Bai YL, Gu SZ. [Comparative study on curative effects of stroke treated with acupuncture by NIRS]. Zhongguo Zhen Jiu. 2011 Nov;31(11):998-1002.
23	Wang L, Liu SM, Liu M, Li BJ, Hui ZL, Gao X. [Post-stroke speech disorder treated with acupuncture and psychological intervention combined with rehabilitation training: a randomized controlled trial]. Zhongguo Zhen Jiu. 2011 Jun;31(6):481-6.
24	Schockert T, Schnitker R, Boroojerdi B, Smith IQ, Yamamoto T, Vietzke K, Kastrau F. Cortical activation by Yamamoto new scalp acupuncture in the treatment of patients with a stroke: a sham-controlled study using functional MRI. Acupunct Med. 2010 Dec;28(4):212-4. doi: 10.1136/aim.2010.002683.
25	Liu JH, Bao CL, Zhu WZ, Zhang GB, Dong GR. [Observation on specificity of acupuncture location in treatment of acute apoplexy by scalp penetration needling]. Zhongguo Zhen Jiu. 2010 Apr;30(4):275-8.
26	Li CF, Jia CS, Li XF, Shi J, Dou ZZ, Sun P. [Effect of penetrative needling of otopoints combined with body acupuncture on limb myodynamia and neurofunction in patients with acute cerebral infarction]. Zhen Ci Yan Jiu. 2010 Feb;35(1):56-60.
27	Li L, Gong JQ, Ding GH, Cai DH, Cai Y. [Effect of multiple paralleled acupuncture needles stimulation of scalp points on hemodynamics and blood flow energy of the common carotid artery in stroke patients]. Zhen Ci Yan Jiu. 2009 Oct;34(5):334-8.
28	Park SU, Shin AS, Jahng GH, Moon SK, Park JM. Effects of scalp acupuncture versus upper and lower limb acupuncture on signal activation of blood oxygen level dependent (BOLD) fMRI of the

	brain and somatosensory cortex. J Altern Complement Med. 2009 Nov;15(11):1193-200. doi: 10.1089/acm.2008.0602.
29	Zhang ZM, Feng CL, Pi ZK, Fan XY, Chen HQ, Zhang J. [Observation on clinical therapeutic effect of acupuncture on upper limb spasticity in the patient of poststroke]. Zhongguo Zhen Jiu. 2008 Apr;28(4):257-60.
30	Zhang HM. [Clinical treatment of apoplectic aphemia with multi-needle puncture of scalp-points in combination with visual-listening-speech training]. Zhen Ci Yan Jiu. 2007;32(3):190-4.
31	Dong JP, Sun WY, Wang S, Wu ZQ, Liu F. [Clinical observation on head point- through-point electroacupuncture for treatment of poststroke depression]. Zhongguo Zhen Jiu. 2007 Apr;27(4):241-4.
32	Yu CD, Wu BH, Zhang J, Song HM, Wang GS, Yu Z. [Effect of skull acupuncture and scalp acupuncture on serum vascular endothelial growth factor in the patient of acute cerebral infarction]. Zhongguo Zhen Jiu. 2006 Jul;26(7):466-8.
33	Lun X, Yang W, Fu B. Effects of CT-localized scalp round-needling on the blood rheology, NO and NOS of patients with multiple infarctional dementia. J Tradit Chin Med. 2006 Jun;26(2):92-6.
34	Sun H, Bao F, Wang DH, Zhang YX, Wang FQ. [Observation on clinical therapeutic effect of scalp acupuncture combined with body acupuncture on apoplectic hemiplegia]. Zhongguo Zhen Jiu. 2006 Jun;26(6):395-8.
35	Li T, Zhao JG, Tian GJ, Zhang L, Liu SJ. [Clinical observation on effect of acupuncture on nervous functions of the patient after operation of. Hypertensive cerebral hemorrhage]. Zhongguo Zhen Jiu. 2006 Apr;26(4):247-9.
36	Kong Y, Xu F, Lin X, Feng Z, Shi H, Yu G, Hu L, Li X, Jiang L. Effects of the lifting manipulation of scalp acupuncture for raising myodynamia of the affected limbs in hemiplegic patients due to cerebral thrombosis. J Tradit Chin Med. 2005 Dec;25(4):256-9.
37	Wang BQ, Zhou P, Zhu YP. [Observation on therapeutic effect of scalp acupuncture combined with body acupuncture on stroke]. Zhongguo Zhen Jiu. 2005 Apr;25(4):240-2.

Supplementary Table 4. Quality assessment of the retrieved studies

Author,Year	Q1	Q2	Q3	Q4	Q5	Q6	Q7	Q8	Q9	Q10	Q11	Q12	Q13	Q14	Total score	Quality Rating
Xie 2007	Y	NR	NR	N	NR	Y	Y	Y	Y	Y	Y	NR	Y	Y	**9/14 (64.3%)**	**Fair**
Li et 2009	Y	Y	N	N	Y	Y	Y	Y	Y	Y	Y	NR	Y	Y	**11/14 (78.6%)**	**Good**
Ma 2010	Y	NR	N	N	NR	Y	Y	Y	Y	Y	Y	NR	Y	Y	**9/14 (64.3%)**	**Fair**
Fu 2011	Y	NR	N	N	NR	Y	Y	Y	Y	Y	Y	NR	Y	Y	**9/14 (64.3%)**	**Fair**
Qin 2013	Y	NR	N	N	N	Y	Y	Y	Y	Y	Y	NR	Y	Y	**9/14 (64.3%)**	**Fair**
Kong 2014	Y	NR	N	N	NR	Y	Y	Y	Y	Y	Y	NR	Y	Y	**9/14 (64.3%)**	**Fair**
Qin D 2015	Y	Y	N	N	N	Y	Y	Y	Y	Y	Y	NR	Y	Y	**10/14 (71.4%)**	**Fair**
Tan 2015	Y	NR	N	N	N	Y	Y	Y	Y	Y	Y	NR	Y	Y	**9/14 (64.3%)**	**Fair**
Xu 2015	Y	NR	N	N	N	Y	Y	Y	Y	Y	Y	NR	Y	Y	**9/14 (64.3%)**	**Fair**
Zhang 2015	Y	Y	N	N	N	Y	Y	Y	Y	Y	Y	NR	Y	Y	**10/14 (71.4%)**	**Fair**
Chen 2016	Y	NR	N	N	NR	Y	Y	Y	Y	Y	Y	NR	Y	Y	**9/14 (64.3%)**	**Fair**
Dou 2016	Y	NR	N	N	N	Y	Y	Y	Y	Y	Y	NR	Y	Y	**9/14 (64.3%)**	**Fair**
Liu 2016	Y	Y	N	N	N	Y	Y	Y	Y	Y	Y	NR	Y	Y	**10/14 (71.4%)**	**Fair**
Pan 2017	Y	NR	N	N	N	Y	Y	Y	Y	Y	Y	NR	Y	Y	**9/14 (64.3%)**	**Fair**
Wang 2017	Y	Y	N	N	Y	Y	Y	Y	Y	Y	Y	Y	Y	Y	**12/14 (85.7%)**	**Good**
Yang 2017	Y	NR	N	N	N	NR	Y	Y	Y	NR	Y	NR	Y	Y	**7/14 (50.0%)**	**Fair**
Yin YH 2017	Y	NR	N	N	N	Y	Y	Y	Y	NR	Y	NR	Y	Y	**8/14 (57.1%)**	**Fair**
Hu HY 2018	Y	NR	N	N	N	Y	Y	Y	Y	NR	Y	NR	Y	Y	**8/14 (57.1%)**	**Fair**
Xiao L 2018	Y	NR	N	N	N	Y	Y	Y	Y	Y	Y	NR	Y	Y	**9/14 (64.3%)**	**Fair**
Xu 2018	Y	N	N	N	N	Y	Y	Y	Y	Y	Y	NR	Y	Y	**9/14 (64.3%)**	**Fair**
Chen 2019	Y	Y	N	N	N	Y	Y	Y	Y	Y	Y	NR	Y	Y	**10/14 (71.4%)**	**Fair**
Hu 2019	Y	NR	N	N	N	Y	Y	Y	Y	Y	Y	NR	Y	Y	**9/14 (64.3%)**	**Fair**
Li 2019	Y	NR	N	N	N	Y	Y	Y	Y	Y	Y	NR	Y	Y	**9/14 (64.3%)**	**Fair**
Ma 2019	Y	Y	N	N	N	Y	Y	Y	Y	Y	Y	NR	Y	Y	**10/14 (71.4%)**	**Fair**
Sun XQ 2019	Y	NR	N	N	N	Y	Y	Y	Y	Y	Y	NR	Y	Y	**9/14 (64.3%)**	**Fair**
Ye 2019	Y	Y	N	N	NR	Y	Y	Y	Y	Y	Y	NR	Y	Y	**10/14 (71.4%)**	**Fair**
Zhang XY 2019	Y	Y	N	N	N	Y	Y	Y	Y	Y	Y	NR	Y	Y	**10/14 (71.4%)**	**Fair**
Zhang HL 2019	Y	Y	N	N	N	Y	Y	Y	Y	Y	Y	NR	Y	Y	**10/14 (71.4%)**	**Fair**
Zhao 2019	Y	NR	N	N	NR	Y	Y	Y	Y	Y	Y	NR	Y	Y	**9/14 (64.3%)**	**Fair**
Zhu 2019	Y	NR	N	N	N	Y	Y	Y	Y	Y	Y	NR	Y	Y	**9/14 (64.3%)**	**Fair**
Qi 2018	Y	Y	N	N	N	Y	Y	Y	Y	Y	Y	NR	Y	Y	**10/14 (71.4%)**	**Fair**
Xiong 2020	Y	Y	N	N	Y	Y	Y	Y	Y	Y	Y	NR	Y	Y	**11/14 (78.6%)**	**Good**
Yin 2020	Y	NR	N	N	N	Y	Y	Y	Y	Y	Y	NR	Y	Y	**9/14 (64.3%)**	**Fair**
Wang 2020	Y	Y	N	N	Y	Y	Y	Y	Y	Y	Y	Y	Y	Y	**12/14 (85.7%)**	**Good**
Zhang 2021	Y	Y	N	N	Y	Y	Y	Y	Y	Y	Y	Y	Y	Y	**12/14 (85.7%)**	**Good**

Quality assessment of the retrieved studies was assessed using the Quality Assessment of Controlled Intervention Studies (National Heart, Lung, and Blood Institute, National Institutes of Health) (www.nhlbi.nih.gov/health-topics/study-quality-assessment-tools).

Caption:
Total Score: Number of yes and the percentage of 14 assessment questions; CD, cannot be determined; NA, not applicable; NR, not reported (light yellow); N, no (light red); Y, yes (light green).

Quality Rating:
Poor <50% (red), Fair 50-75% (green), Good ≥75% (blue).

Assessment questions:
Q1. Was the study described as randomized, a randomized trial, a randomized clinical trial, or an RCT?
Q2. Was the method of randomization adequate (i.e., use of randomly generated assignment)?
Q3. Was the treatment allocation concealed (so that assignments could not be predicted)?
Q4. Were study participants and providers blinded to treatment group assignment?
Q5. Were the people assessing the outcomes blinded to the participants' group assignments?
Q6. Were the groups similar at baseline on important characteristics that could affect outcomes (e.g., demographics, risk factors, co-morbid conditions)?
Q7. Was the overall drop-out rate from the study at endpoint 20% or lower of the number allocated to treatment?
Q8. Was the differential drop-out rate (between treatment groups) at endpoint 15 percentage points or lower?
Q9. Was there high adherence to the intervention protocols for each treatment group?
Q10. Were other interventions avoided or similar in the groups (e.g., similar background treatments)?
Q11. Were outcomes assessed using valid and reliable measures, implemented consistently across all study participants?
Q12. Did the authors report that the sample size was sufficiently large to be able to detect a difference in the main outcome between groups with at least 80% power?
Q13. Were outcomes reported or subgroups analyzed prespecified (i.e., identified before analyses were conducted)?
Q14. Were all randomized participants analyzed in the group to which they were originally assigned, i.e., did they use an intention-to-treat analysis?

PRISMA 2020 Checklist

Section and Topic	Item #	Checklist item	Location where item is reported
**TITLE**			
Title	1	Identify the report as a systematic review.	P1
**ABSTRACT**			
Abstract	2	See the PRISMA 2020 for Abstracts checklist.	P1
**INTRODUCTION**			
Rationale	3	Describe the rationale for the review in the context of existing knowledge.	P2
Objectives	4	Provide an explicit statement of the objective(s) or question(s) the review addresses.	P3
**METHODS**			
Eligibility criteria	5	Specify the inclusion and exclusion criteria for the review and how studies were grouped for the syntheses.	P3 and Supplementary Table 3
Information sources	6	Specify all databases, registers, websites, organisations, reference lists and other sources searched or consulted to identify studies. Specify the date when each source was last searched or consulted.	P3 and Supplementary Table 1
Search strategy	7	Present the full search strategies for all databases, registers and websites, including any filters and limits used.	P3 and Supplementary Table 1
Selection process	8	Specify the methods used to decide whether a study met the inclusion criteria of the review, including how many reviewers screened each record and each report retrieved, whether they worked independently, and if applicable, details of automation tools used in the process.	P3
Data collection process	9	Specify the methods used to collect data from reports, including how many reviewers collected data from each report, whether they worked independently, any processes for obtaining or confirming data from study investigators, and if applicable, details of automation tools used in the process.	P3
Data items	10a	List and define all outcomes for which data were sought. Specify whether all results that were compatible with each outcome domain in each study were sought (e.g. for all measures, time points, analyses), and if not, the methods used to decide which results to collect.	P3

Section and Topic	Item #	Checklist item	Location where item is reported
	10b	List and define all other variables for which data were sought (e.g. participant and intervention characteristics, funding sources). Describe any assumptions made about any missing or unclear information.	P3
Study risk of bias assessment	11	Specify the methods used to assess risk of bias in the included studies, including details of the tool(s) used, how many reviewers assessed each study and whether they worked independently, and if applicable, details of automation tools used in the process.	P3-4
Effect measures	12	Specify for each outcome the effect measure(s) (e.g. risk ratio, mean difference) used in the synthesis or presentation of results.	P3
Synthesis methods	13a	Describe the processes used to decide which studies were eligible for each synthesis (e.g. tabulating the study intervention characteristics and comparing against the planned groups for each synthesis (item #5)).	N/A
	13b	Describe any methods required to prepare the data for presentation or synthesis, such as handling of missing summary statistics, or data conversions.	N/A
	13c	Describe any methods used to tabulate or visually display results of individual studies and syntheses.	N/A
	13d	Describe any methods used to synthesize results and provide a rationale for the choice(s). If meta-analysis was performed, describe the model(s), method(s) to identify the presence and extent of statistical heterogeneity, and software package(s) used.	N/A
	13e	Describe any methods used to explore possible causes of heterogeneity among study results (e.g. subgroup analysis, meta-regression).	N/A
	13f	Describe any sensitivity analyses conducted to assess robustness of the synthesized results.	N/A
Reporting bias assessment	14	Describe any methods used to assess risk of bias due to missing results in a synthesis (arising from reporting biases).	N/A
Certainty assessment	15	Describe any methods used to assess certainty (or confidence) in the body of evidence for an outcome.	N/A
		**RESULTS**	
Study selection	16a	Describe the results of the search and selection process, from the number of records identified in the search to the number of studies included in the review, ideally using a flow diagram.	P4 and Figure 1

Section and Topic	Item #	Checklist item	Location where item is reported
	16b	Cite studies that might appear to meet the inclusion criteria, but which were excluded, and explain why they were excluded.	Supplementary Table 3
Study characteristics	17	Cite each included study and present its characteristics.	P4 and Table 1
Risk of bias in studies	18	Present assessments of risk of bias for each included study.	P6 and Supplementary Table 4
Results of individual studies	19	For all outcomes, present, for each study: (a) summary statistics for each group (where appropriate) and (b) an effect estimate and its precision (e.g. confidence/credible interval), ideally using structured tables or plots.	Table 1 and Table 2
Results of syntheses	20a	For each synthesis, briefly summarise the characteristics and risk of bias among contributing studies.	N/A
	20b	Present results of all statistical syntheses conducted. If meta-analysis was done, present for each the summary estimate and its precision (e.g. confidence/credible interval) and measures of statistical heterogeneity. If comparing groups, describe the direction of the effect.	N/A
	20c	Present results of all investigations of possible causes of heterogeneity among study results.	N/A
	20d	Present results of all sensitivity analyses conducted to assess the robustness of the synthesized results.	N/A
Reporting biases	21	Present assessments of risk of bias due to missing results (arising from reporting biases) for each synthesis assessed.	N/A
Certainty of evidence	22	Present assessments of certainty (or confidence) in the body of evidence for each outcome assessed.	N/A
**DISCUSSION**			
Discussion	23a	Provide a general interpretation of the results in the context of other evidence.	P6-10
	23b	Discuss any limitations of the evidence included in the review.	P10
	23c	Discuss any limitations of the review processes used.	P10
	23d	Discuss implications of the results for practice, policy, and future research.	P10
**OTHER INFORMATION**			
Registration and protocol	24a	Provide registration information for the review, including register name and registration number, or state that the review was not registered.	N/A

Section and Topic	Item #	Checklist item	Location where item is reported
	24b	Indicate where the review protocol can be accessed, or state that a protocol was not prepared.	N/A
	24c	Describe and explain any amendments to information provided at registration or in the protocol.	N/A
Support	25	Describe sources of financial or non-financial support for the review, and the role of the funders or sponsors in the review.	P10
Competing interests	26	Declare any competing interests of review authors.	P10
Availability of data, code and other materials	27	Report which of the following are publicly available and where they can be found: template data collection forms; data extracted from included studies; data used for all analyses; analytic code; any other materials used in the review.	P10
